# Giant Benign Struma Ovarii with High-Grade Fever, Elevated CA 125, and Hormonal Function in an Adolescent Patient

**DOI:** 10.3390/children10050856

**Published:** 2023-05-11

**Authors:** Ioana Anca Stefanopol, Alexandru Petecariu, Liliana Baroiu, Anca-Iulia Neagu, Roxana-Elena Bogdan-Goroftei, Alexandru Nechifor, Diana-Andreea Ciortea, Nicolae Sarbu

**Affiliations:** 1 Clinical Surgical Department, Faculty of Medicine and Pharmacy, “Dunărea de Jos” University, 800216 Galați, Romania; ancaflorea1969@yahoo.com (I.A.S.); ancazanoschi@gmail.com (A.-I.N.); 2 Department of Pediatric Surgery, “Sf Ioan” Clinical Emergency Hospital for Children, 800487 Galați, Romania; 3 Clinical Medical Department, Faculty of Medicine and Pharmacy, “Dunărea de Jos” University, 800216 Galați, Romania; 4 Infectious Diseases Department, “Sf Cuv Parascheva” Clinical Hospital of Infectious Diseases, 800179 Galați, Romania; 5 Department of Anatomopathology, “Sf Ioan” Clinical Emergency Hospital for Children, 800487 Galați, Romania; 6 Emergency Department, “Sf Ioan” Clinical Emergency Hospital for Children, 800487 Galați, Romania; 7 Pediatric Department, “Sf Ioan” Clinical Emergency Hospital for Children, 800487 Galați, Romania; 8 Faculty of Medicine and Pharmacy, “Dunărea de Jos” University, 800216 Galați, Romania; 9 Department of Radiology and Medical Imaging, “Sf Ioan” Clinical Emergency Hospital for Children, 800487 Galați, Romania

**Keywords:** struma ovarii, ovarian cystadenoma, treatment, adolescent, CA 125, infected ovarian cyst

## Abstract

Struma ovarii (SO) is a monodermal teratoma containing at least 50% thyroid tissue. Classically, SO is a hormonally inactive benign neoplasm that occurs in premenopausal women, and has unspecific clinical and imaging features. Its treatment is surgical and its diagnosis is established histopathologically. We report the case of a euthyroid 16-year-old girl presenting with abdominal girth increase. An abdomino-pelvic ultrasound showed a giant multicystic mass with transonic content and multiple septa, and magnetic resonance imaging suggested the diagnosis of right ovarian mucinous cystadenoma. Blood tests showed inflammatory syndrome, iron deficiency anemia, mild hepatocytolysis, and elevated serum CA 125 levels. High-grade fever occurred on the third day of hospitalization, but none of the preoperative tests could identify its origin. Cystectomy was performed, and the histopathological examination revealed benign SO with a few small cysts with purulent content. The patient developed hypothyroidism postoperatively. In conclusion, this case report reunites most of the uncommon features of SO and confirms the superiorityof histopathology in its definitive diagnosis, as well as the suitability of ovarian sparing techniques, as the best treatment option for cystic ovarian pathology in pediatric patients, even in cases of large tumoral size and elevated serum CA 125 levels.

## 1. Introduction

Struma ovarii (SO) is a monodermal teratoma containing at least 50% thyroid tissue [1]. Data from the literature show that it represents 2–3% of ovarian teratomas and 1% of all ovarian tumors [2]. Although mature cystic teratomas represent up to 70% of the benign ovarian neoplasms in pediatric patients [3], SO occurs most often in premenopausal women, and is uncommon in children and adolescents. SO usually arises unilaterally and in the left ovary [4]. This neoplasm is most often benign, of low malignancy, and of low metastatic potential [5]. Malignant transformation may occur in less than 5% of cases, with papillary carcinoma as the most frequent histological type [6].

The clinical features of SO are non-specific, the main symptoms being abdominal pain and abdominal girth increase [7]. In very rare cases, serum levels of CA 125 may be elevated, leading the surgeon to draw an incorrect conclusion about the malignant nature of the ovarian tumor [8]. The classical SO is non-functional, but in 5–12% of cases, it can be hormonally active [9].

Regardless of the imaging technique used, SO’s aspect is indistinct and often overlaps with other ovarian lesions, or even with ovarian malignancy [10]. The multiloculated cystic component is always present, but 73% of benign SO cases also contain solid components [11]. However, “struma pearls” on ultrasound (US) imaging seem to have some specificity [12], as well as strong enhancement of the solid components with a lacy pattern on magnetic resonance imaging (MRI) after gadolinium injection [13].

SO treatment is surgical, but there are no consistent data on its management protocol. Regarding the extent of removal, it is worth mentioning that the final decision depends on whether the appearance of the tumor is benign or malignant, as well as on the patient’s age, her potential for future fertility, and even her own decision [4]. The definitive diagnosis usually relies on histopathological examination [10].

We present the case of an euthyroid 16-year-old female patient with a giant benign SO, high-grade fever, and elevated serum CA 125 levels, who underwent cystectomy and developed hypothyroidism postoperatively. Written informed consent for publication of this case report and the accompanying images was obtained from the patient’s mother. 

## 2. Case Report

**Figure 1:**
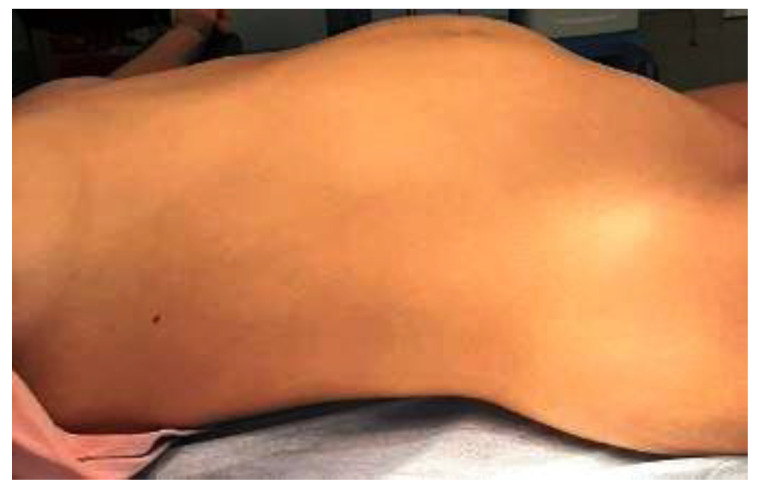
Clinical aspect: increase in abdominal girth due to a giant palpable abdominal mass.

A 16-year-old girl from an urban area was presented to the emergency room of our hospital. A week before, she had started a symptomatic treatment with ibuprofen for low-grade fever and slight cough, without medical advice. Since the fever persisted, she went to a general physician, who noticed an increase in the abdominal girth due to a giant palpable abdominal mass and recommended an urgent visit to the hospital. She was admitted to the department of pediatric surgery, and we found that the progressive abdominal enlargement had started about a year prior, and was associated with intermittent mild pain. The patient had no medical history, had her menarche at 12 years old, and had normal menstrual cycles. Upon admission, she was asymptomatic with normal body temperature, and the clinical examination revealed a giant, tense, and relatively mobile tumor with a regular surface, and umbilical hernia. Additionally, in the right thoracic area, a brown-black lesion was observed (Figure 1). 

**Figure 2:**
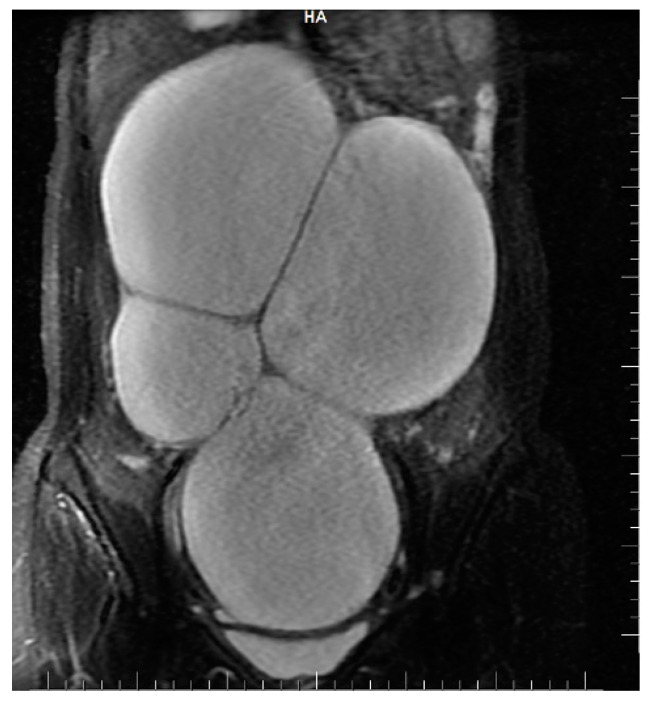
Coronal STIR sequence shows a well-defined voluminous, multiloculated, multiseptate cystic mass occupying the pelvis and the large peritoneal cavity.

**Figure 3:**
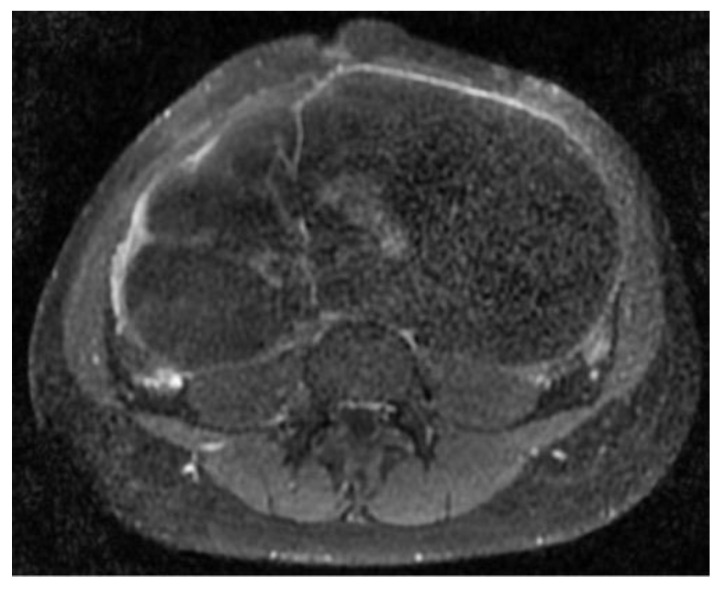
Axial postcontrast T1 sequence shows a cystic mass with moderate mural and septate contrast enhancement.

The abdomino-pelvic US showed a thin-walled, multilocular cystic mass occupying almost the entire abdomen and pelvis, with transonic content and multiple septa, some of them thick. None of the ovaries were visualized, and there was no free peritoneal fluid. Because the cyst origin could not be specified, we requested an abdomino-pelvic MRI, which showed a 115/224/350 mm cystic tumor originating in the right ovary that waswell-defined by a regular wall, multiloculated, had liquid content with some areas of high signal intensity in T1, and presented moderate mural and septal enhancement after gadolinium injection; the suggested diagnosis was ovarian cystadenoma (Figure 2 and Figure 3).

The patient’s blood tests presented some abnormal values: hemoglobin: 10.12 g/dL (12–15 g/dL), hematocrit: 29.4% (35–45%), thrombocytes: 656.4 × 10^3^/µL (150–450 × 10^3^/µL), C-reactive protein: 26.45 mg/dL (0–0.5 mg/dL), D-dimers: 1.21 mg/L (0–0.55 mg/L), alkaline phosphatase: 213 U/L (50–130 U/L), ferritin: 480 ng/mL (13–68 ng/mL), iron: 28 µg/dL (37–170 µg/dL), aspartate aminotransferase: 70 U/L (14–36 U/L), alanine aminotransferase: 77 U/L (4–44 U/L), and gamma-glutamil-transpeptidase: 353 U/L (11–28 U/L). At the time of the study, we requested a pediatric endocrinological consultation for all adnexal lesions, and in this case, the hormonal tests were normal, including those for thyroid function. The carcinoembryonic antigen (CEA), α fetoprotein (AFP), β human chorionic gonadotropin (HCG), LDH, and CA 19-9 values were within the normal limits, but CA 125 was increased to 124.7 U/mL (<30.2 U/mL).

**Figure 4:**
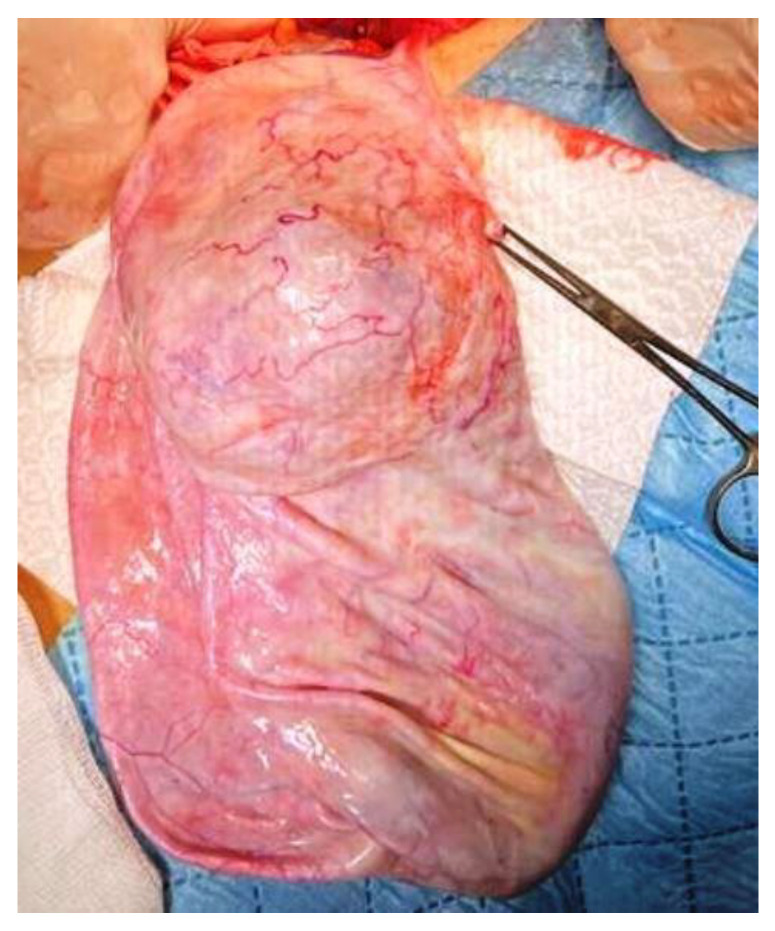
Ovarian cyst after drainage of 4500 mL clear colorless liquid, and the 40 mm non-communicating cyst. Some small cysts within the walls can also be seen.

**Figure 5:**
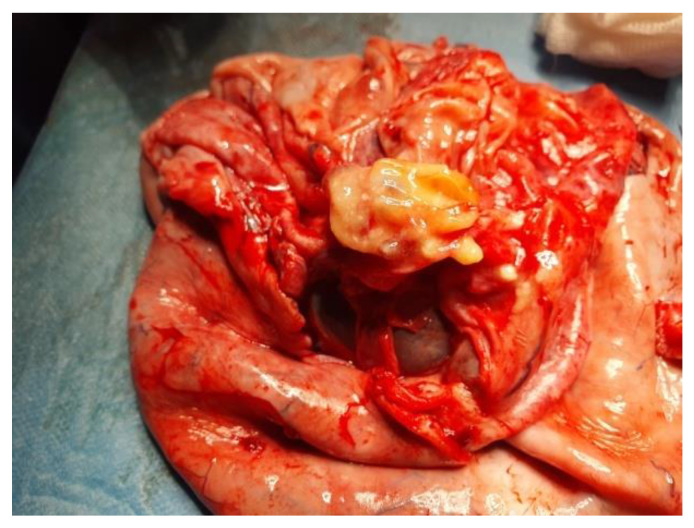
The well-defined 20 mm solid round yellow component with fat-like content, and 2 small cysts with brownish content.

Given the inflammatory syndrome, the iron deficiency anemia, and the mild hepatocytolysis, the preanesthetic examination was followed by additional investigations and preoperatory treatment with Ceftriaxone. Uroculture, as well as the nasal and pharyngeal exudate, did not detect any pathogenic microorganisms, and the pulmonary radiography was normal. On the third day of hospitalization, the patient presented high-grade fever (up to 39.5 °C) that was unresponsive to an intravenous antibiotic and antipyretic treatment, and was unaccompanied by other symptoms. We excluded the usual causes of fever, such as otorhinolaryngeal, respiratory, digestive, urinary, or cutaneous conditions, and the hemoculture and the ARN SARS-CoV-2 test were negative. We thought of an intracystic hemorrhage or infection, but the cyst content was unchanged on the abdominal US. A pediatric oncological examination raised the suspicion of a paraneoplastic syndrome and sepsis, and prompted intravenous treatment with meropenem, gentamicin, and metronidazole. After 2 days, the patient’s body temperature became normal. Consequently, with a presumptive preoperatory diagnosis of serous or mucinous cystadenoma, we performed a 3 cm subumbilical median incision. The cyst was underlying the abdominal wall, and drainage was performed under direct visualization. Because the sudden decompression of a giant cyst may lead to some changes in respiration and circulation due to an increase in venous return, we initiated slow drainage by using a puncture needle for the first 1000 mL, followed by a high-pressure suction cannula, without any spillage. In total, 4500 mL of clear, colorless liquid was extracted. A 40 mm cyst remained undrained, due to the fact that it did not communicate with the rest of the tumor (Figure 4). We performed cystectomy, preserving the ovary. After complete removal of the tumor, inside the bottom of the cyst, near the ovary, we found a well-defined 20 mm solid round yellow component, with fat-like content. We also determined some small cysts with brownish content (Figure 5). A final inspection of the abdomen and the pelvis revealed no further issues. We closed the incision and performed an umbilical hernia repair. The patient had an uneventful postoperative evolution and was discharged after 3 days.

**Figure 6:**
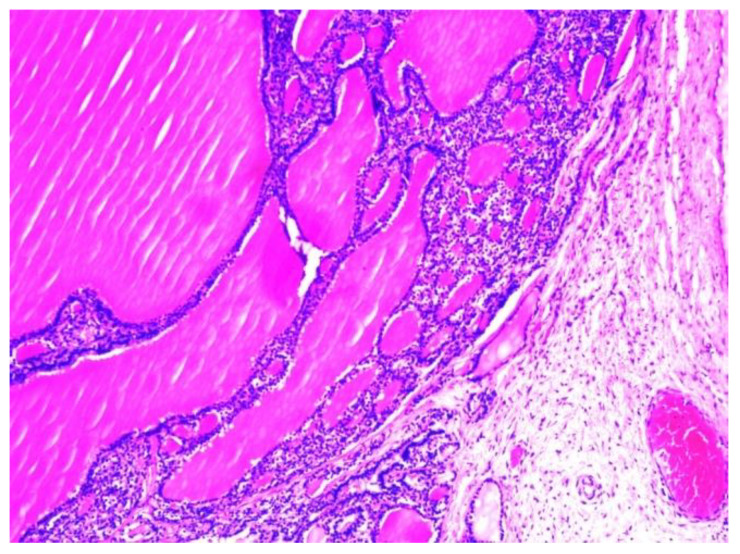
Histopathological aspect of the cystic wall, with variable-sized mature thyroid follicles filled with colloid (H&E stain, magnification: 100×).

**Figure 7:**
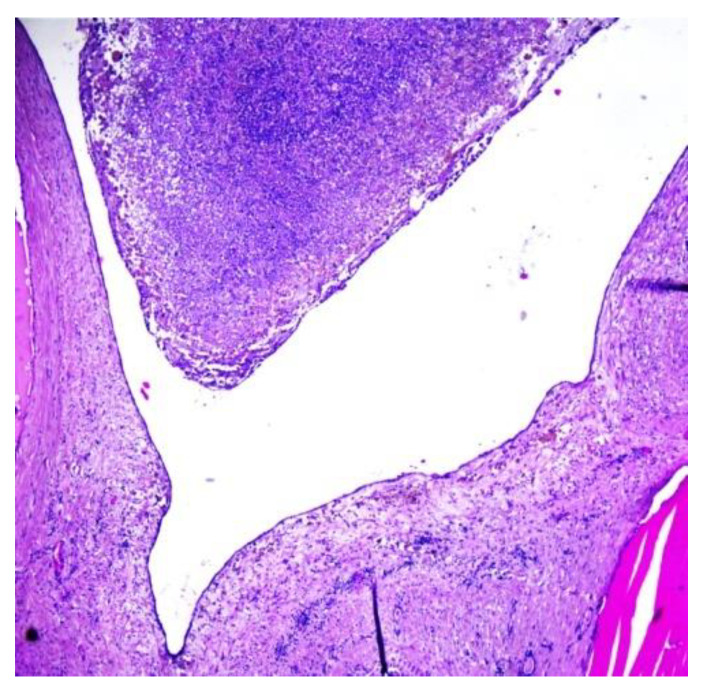
Histopathological aspect of a cyst lined withflat epithelium, containing purulent exudate (H&E stain, magnification: 40×).

The histopathological examination of the multilocular cystic mass was consistent with benign SO, as its walls were composed of mature thyroid tissue without architectural or cytological atypia, with variable-sized follicles containing eosinophilic colloid (Figure 6). The stroma between the follicles was fibrous with focal extracellular cholesterol deposits. The largest cyst was lined withflat epithelium, with some thyroid follicles in the fibrous walls. The content of some small cysts was represented by purulent exudate (Figure 7). No other epithelia were detected. 

Since the patient had normal blood tests for thyroid function before surgery, the histopathological findings required us to perform an endocrinological re-evaluation. This time, the biochemical features showed subclinical hypothyroidism (TSH: 4.36 µUI/mL; normal values: 0.27–4.20 µUI/mL) with a normal thyroid on the US, and daily hormone replacement therapy with levothyroxine was recommended.

## 3. Discussion

Although thyroid cells may be found in about 20% of dermoid cysts, SO is a monodermal mature ovarian teratoma that is histopathologically defined by finding thyroid follicular tissue in more than 50% of the tumor [6]. Like all teratomas, which grow slowly by about 1.8 mm each year [14], SO also takes years to develop. Although it is considered that SO is usually smaller than 100 mm, there have also been reported cases of larger tumors. Except for the current case (350 mm), we found only one other SO measuring 300 mm, in a patient of the same age as ours [1].

Considered to occur mainly in women aged between 30 and 50 years [15], SO is certainly not characteristic of pediatric patients [16]. To the best of our knowledge, so far, there have only been four reported cases of benign SO in pediatric patients, who were aged 6, 14, 16, and 17 years [1,16,17]. Moreover, studies carried out on larger groups of patients did not report children or adolescents [2,5,18].

Up to 40% of patients are asymptomatic, and the tumor is discovered incidentally during imaging examination. When present, the most common complaints are abdominal pain and distension [4,9], usually controlled with non-steroidal anti-inflammatory drugs and COX-2 inhibitors, which cause well-known adverse reactions [19,20]. If the tumor reaches a large size, signs of neighboring organ compression may occur, as well, such as: dysuria, polyuria or hydronephrosis, constipation, etc. [21]. Abdominal enlargement is commonly determined by a palpable pelvic or abdominopelvic mass, as was the case in our patient, but in one-third of cases, it may be due to the associated ascites. Regardless of age, finding a giant ovarian mass necessitates higher awareness and further investigation, such as imaging and the assessment of serum tumoral markers. Although CA 125 is known as a predictive factor ofthe malign nature of an ovarian mass, some authors have described elevated levels of this marker in patients with benign tumors [22]. Elevated serum CA 125 levels associated with SO have rarely been reported, and this is considered a secondary effect due to ascites rather than a direct consequence of tumor presence [2]. Whenever this situation occurs, it may lead to inappropriate aggressive management [23]. Other possible symptoms are: dyspnoea, vaginal bleeding, tachycardia, and deep vein thrombosis [7,9]. 

Regarding thyroid hormone secretion, SO can be inactive or active; recent studies have shownthat SO is the only serum thyroglobulin-secretingtumor [4]. When active, patients may present with three types of clinical feature: common signs of hyperthyroidism with origins in both the thyroid gland and SO; thyrotoxicosis with a normal functional thyroid gland, which is the only circumstance in which SO may be diagnosed preoperatory; and less often, as in the above-presented case, a euthyroid patient who becomes hypothyroid after surgery because the ectopic thyroid tissue within the ovary is the only functional thyroid tissue [4,9]. 

The most common pattern on a US is a multilocular cystic mass with a lobulated surface, multiple cysts (dilated thyroid follicles), thickened septa, and various amounts of solid components (thyroid tissue). The cyst fluid is always anechoic or of low-level echogenicity. Because the thyroid tissue is highly vascular, upon color Doppler examination, most SO cases appear moderately vascularized and display variable flow, ranging from none to abundant. A typical feature of struma ovarii is “struma pearls”, which consist of one or more round, solid areas that are well-circumscribed by a smooth margin, and well vascularized upon Doppler examination. On the other hand, most often, these images are considered papillary projections, raising the suspicion of malignancy [11,24,25]. Other US patterns have been described: a solid tumor with heterogeneous internal echogenicity and cystic spaces, or, as in the case of our patient, a multilocular cystic mass without visible solid components misleading to a follicular cyst or a benign cystic ovarian neoplasm [26,27].

MRI is a more accurate method of identifying the nature of cystic content. The typical appearance of SO on T1-weighted images (T1WIs) shows a multilocular cystic lesion with loculi that have variable signal intensities and punctuate foci of high signal intensity, located inside or adjacent to the thickened septa or the cyst walls. T1WIs show strong enhancement of the solid components after gadolinium injection. In T2-weighted sequences, most loculi have high signal intensity. There is no enhancement of these loculi on fat-saturated postcontrast T1WI sequences [25,26].

Except for cases in which the patient has hyperthyroidism and a normal thyroid, it may be inappropriate to speak of a differential diagnosis for SO, since this extremely rare condition is not usually the first preoperative diagnostic choice, and is often misdiagnosed as mucinous or serous cystadenoma [15], a functional cyst, or even a malignant neoplasm (in the presence of an important solid component). Sometimes, SO can have imaging aspects similar to hydrosalpinx, a tubo-ovarian abscess, an ectopic pregnancy, or endometrioma [7,8].

The histopathologic examination of anovarian mass establishes a definitive diagnosis by revealing mature thyroid follicles of various sizes, lined by cuboidal epithelial cells and containing colloid [10,12]. If necessary, the diagnosis of SO can be confirmed via animmunohistochemistry test for thyroglobulin [4].

Despite being described for the first time in 1895 by Von Klden, and in 1899 by Gottschalk [5,16], there still is not a consensus regarding the opportunity for conservative or radical surgery. Certainly, the only way to manage this tumor is through its surgical removal, and since the probability of a correct preoperative diagnosis is minimal, the major role of surgery is to enable a definitive histopathological diagnosis, and thus, to establish proper postoperative care. Based on the frequently benign nature of SO, and on its low metastatic potential and low risk of recurrence, recent studies advise a more conservative approach consisting of cystectomy only [5]. Our patient had a nevi on her thorax, with no other skin malignancies or metastasis, but some recent papers have shown that in exceedingly rare cases, distant metastases inthe lungs, bones, liver, skin, and brain may appear [28,29]. However, even for benign SO, there are authors who advise salpingo-oophorectomy [10,15]. On the other hand, total hysterectomy with bilateral salpingo-oophorectomy can occasionally be indicative in postmenopausal women [4,8].

The postoperative follow-up for benign SO consist mainly of an ultrasound and thyroid function investigations [5]. Some authors consider that the immediate postoperative course is sufficient [1], but others recommend yearly monitoring while taking into account the possibility of recurrence, even after more than 10 years [15].

Our patient was admitted with a giant cystic multiloculated abdominal tumor appearing on an ultrasound, and because we had no information on its origin, the initial differential diagnosis included abdominal lymphangioma and ovarian cystadenoma. We also took into consideration a paraovarian or follicular cyst. The MRI showed the ovarian origin of the mass. Corroborating the clinical and imaging features, we were sure it was a mucinous rather than a serous cystadenoma: a benign tumor, that can grow to gigantic sizes, is multiseptated, and has high-signal-intensity locules on T1WI [27]. Since the patient had neither ascites nor pleural effusion, we excluded Meigs or pseudo-Meigs syndrome. We planned a laparoscopic cystectomy, but elevated CA 125 levels and a high fever of unidentified cause led us to suspect a paraneoplastic syndrome and prompted us and the patient’s parents to choose the open technique. Despite the fact that there were a few malignancy criteria(large tumoral size, elevated CA 125), we performed an ovarian sparing technique, considering the girl’s age and the low likelihood of ovarian malignancy in pediatric patients. Finally, the cyst proved to be a functional benign SO, and its excision triggered hypothyroidism. An unsolved problem still remained: what caused the fever? We considered the small solid component with fat-like content, found in the cyst’s wall, to be a coexistent dermoid cyst. Consequently, we did not think to take tissue cultures. Based on the histopathological findings of the purulent exudate, we concluded that the preoperative fever was probably a consequence of cyst infection, an extremely rare complication of ovarian cysts. Azami et al. reported a case of an ovarian dermoid cyst with super-infection caused by Schistosoma hematobium, but the blood cell count was normal and the C-reactive protein level was at 290 mg/L [30]. Currently, 6 months have passed since the operation, and the patient has shown no recurrence on the US and still needs hormonal treatment. The next follow-up will consist of an abdomino-pelvic ultrasound in another 6 months, and yearly thereafter. As concerns thyroid function, its surveillance will be the responsibility of an endocrinologist.

## 4. Conclusions

This case report reunites most of the uncommon features of SO, a very uncommon ovarian tumor: the young age of the patient, the giant size of the SO, the association of the SO with elevated serum CA 125 levels without ascites, the absence of “struma pearls”, the postoperative hypothyroidism triggered by a hormonally active type of tumor, and the cyst infection, as a complication. In addition, it confirms histopathology’s superiority in establishing a definitive SO diagnosis, and the suitability of the ovarian sparing techniques as the best treatment option for cystic ovarian pathology in pediatric patients, even in cases of large tumoral size and elevated serum CA 125 levels.

## Data Availability

The datasets are available from the corresponding author upon reasonable request.

## Abbreviations

SO: struma ovari; US: ultrasound; MRI: magnetic resonance imaging; CEA: carcinoembryonic antigen; AFP: α fetoprotein; β HCG: β human chorionic gonadotropin; H&E: hematoxylin eosinophil; T1WI: T1-weighted images.
